# Correlation between BDNF levels and folic acid levels at baseline in drug-naïve First Episode Psychosis

**DOI:** 10.1192/j.eurpsy.2023.442

**Published:** 2023-07-19

**Authors:** A. Toll, D. Bergé, I. Canosa, M. Martín - Subero, T. Legido, C. Fernandez - Hinchado, V. Perez - Sola, A. Mané

**Affiliations:** ^1^ Institut de Neuropsiquiatria i Addiccions (INAD), Parc de Salut Mar; ^2^Mental Health Group, Institut Mar d’Investigacions Médiques (IMIM); ^3^G21, CIBERSAM; ^4^ Institut de Neuropsiquiatria i Adiccions (INAD), Parc de Salut Mar; ^5^Mantal Health Group, Institut Mar d’Investigacions Médiques (IMIM), Barcelona; ^6^ESM Rural, Montijo - Puebla de la Calzada, Spain

## Abstract

**Introduction:**

Schizophrenia is a severe and common psychiatric disorder characterized by disturbed brain development. Brain-derived neurotrophic factor (BDNF) mediates differentiation and survival of neurons as well as synaptic plasticity during the brain development. Several studies have shown decreased serum levels of BDNF in chronic, first episode, and drug naïve schizophrenia patients. Folate provides the substrate for intracellular methylation reactions that are essential to normal brain development and function. Abnormal folate metabolism has been implicated in schizophrenia. For example, reduced maternal folate intake associated with an increased risk for schizophrenia. Also, low blood levels of folate have been reported in patients with schizophrenia, and are associated with clinical manifestation especially in the negative symptom domain.

**Objectives:**

With this study, we want to know how BDNF levels at baseline in *drug-naïve* FEP are associated with folic acid.

**Methods:**

Fifty *drug-naïve* FEP treated between April 2013 and July 2017 at the ETEP Program at Hospital del Mar were included. Inclusion criteria were: 1) age 18-35 years; 2) DSM-IV-TR criteria for brief psychotic disorder, schizophreniform disorder, schizophrenia or unspecified psychosis; 3) no previous history of severe neurological medical conditions or severe traumatic brain injury; 4) presumed IQ level > 80, and 5) no substance abuse or dependence disorders except for cannabis and/or nicotine use. All patients underwent an assessment at baseline including sociodemographic and clinical variables. Fasting blood samples were obtained before administering any medication at baseline and used to determine folic acid and BDNF levels.

**Results:**

In our *drug-naïve* FEP sample, folic acid levels showed a significative positive correlation with BDNF levels at baseline (r = 0.584; p = 0.003). Moreover, we did a lineal regression model that showed that the baseline variables that better predict BDNF levels were folic acid levels, and cannabis use.

**Conclusions:**

Our results are consistent with the findings from some of previous studies that also shows that lower folic acid levels are associated with lower BNDF levels at baseline in *drug-naïve* FEP. Folate deficiency is associated with cerebrovascular and neurological diseases, and mood disorders. The importance of folate in the nervous system was initially demonstrated in studies that established a greatly increased risk of neurodevelopmental disorders in the offspring of folate-deficient pregnant women. In the adult, epidemiological studies have linked lack of folate to neurodegenerative and neuropsychiatric diseases. However, the mechanisms by which chronic folate deficiency adversely affects CNS function are incompletely understood. Some studies in animals models have hypothesized that folate deficiency in animals could be associated with pyramidal cell loss and reduced hippocampal BDNF.

**Disclosure of Interest:**

None Declared

